# A legal dispute resolution intervention for patients with substance use disorders: a study protocol for a randomized controlled trial

**DOI:** 10.1186/s12889-023-15296-5

**Published:** 2023-03-06

**Authors:** Meghan M. O’Neil, Rebecca A. Johnson, David Córdova, Jenna Pryor, Debra A. Pinals

**Affiliations:** 1 Institute for Social Research, Population Studies Center, University of Michigan, 426 Thompson Street, Ann Arbor, MI 48104 USA; 2 Georgetown University, McCourt School of Public Policy, 37th and O Streets NW, Washington, DC 20057 USA; 3 School of Social Work, University of Michigan, 1080 S. University, Ann Arbor, MI 48109 USA; 4 Department of Psychiatry, University of Michigan, Rachel Upjohn Building, 4250 Plymouth Rd, Ann Arbor, MI 48109 USA

**Keywords:** Substance use disorder, Opioids, Alcohol / Alcoholism, Polysubstance, Legal, Criminal, Justice, Social determinants of health, African American, American Indian Alaska Native (AIAN), Medicaid

## Abstract

**Background:**

Substance use disorders (SUDs) represent major public health concerns and are linked to enhanced risk of legal consequences. Unresolved legal issues may prevent individuals with SUD from completing treatment. Interventions aimed at improving SUD treatment outcomes are limited. Filling that gap, this randomized controlled trial (RCT) tests the ability of a technology-assisted intervention to increase SUD treatment completion rates and improve post-treatment health, economic, justice-system, and housing outcomes.

**Methods:**

A randomized controlled trial with a two-year administrative follow-up period will be conducted. Eight hundred Medicaid eligible and uninsured adults receiving SUD treatment will be recruited at community-based non-profit health care clinics in Southeast, Michigan, USA. Using an algorithm embedded in a community-based case management system, we randomly assign all eligible adults to one of two groups. The treatment/intervention group will receive hands-on assistance with a technology aimed at resolving unaddressed legal issues and the control group receives no treatment. Upon enrollment into the intervention, both treatment (*n* = 400) and control groups (*n* = 400) retain traditional options to resolve unaddressed legal issues, such as hiring an attorney, but only the treatment group is targeted the technology and offered personalized assistance in navigating the online legal platform. To develop baseline and historical contexts for participants, we collect life course history reports from all participants and intend to link those in each group to administrative data sources. In addition to the randomized controlled trial (RCT), we used an exploratory sequential mixed methods and participatory-based design to develop, test, and administer our life course history instruments to all participants. The primary objective is to test whether targeting no-cost online legal resources to those experiencing SUD improves their long-term recovery and decreases negative health, economic, justice-system, and housing outcomes.

**Discussion:**

Findings from this RCT will improve our understanding of the acute socio-legal needs faced by those experiencing SUD and provide recommendations to help target resources toward the areas that best support long-term recovery. The public health impact includes making publicly available a deidentified, longitudinal dataset of uninsured and Medicaid eligible clients in treatment for SUD. Data include an overrepresentation of understudied groups including African American and American Indian Alaska Native persons documented to experience heightened risk for SUD-related premature mortality and justice-system involvement. Within these data, several intended outcome measures can inform the health policy landscape: (1) health, including substance use, disability, mental health diagnosis, and mortality; (2) financial health, including employment, earnings, public assistance receipt, and financial obligations to the state; (3) justice-system involvement, including civil and criminal legal system encounters; (4) housing, including homelessness, household composition, and homeownership.

**Trial registration:**

Retrospectively registered # NCT05665179 on December 27, 2022.

## Background

### Substance use disorders and unresolved legal issues

Drug overdose is now the leading cause of death for American adults under the age of 50, making it one of America’s most important public policy crises of the decade [1]. Mortality is high among individuals with a substance use disorder (SUD) and mortality rates for opioid users are reported to be 6–20 times higher than that of other Americans [2]. Individuals with SUD have low rates of recovery [3], and among opioid users, long-term abstinence rates are less than 30%^1^ and there are high rates of subsequent dependence on alcohol and other substances for those who do recover from opioid misuse [2]. While other research studies medical interventions to help remove barriers to recovery [4–9], qualitative research shows that those in recovery face a complex set of social and structural barriers to reducing their dependence on substances [10, 11].

One set of structural barriers that those in recovery face are continued entanglements with and surveillance from the U.S. criminal and civil justice systems. Individuals with SUD are disproportionately involved in the justice system, with 4 out of 5 defendants on criminal dockets estimated to struggle with SUD [12]. Civil court dockets focused on issues like traffic violations, custody disputes, homeless citations, foreclosures, and evictions disproportionately involve households struggling with SUD [13]. Justice system involvement has two sets of consequences. First are threats to the person’s liberty—e.g., parole supervision that can send a defendant back to incarceration for substance-related violations [14]. Second are what we call “unresolved legal issues.” Unresolved legal issues span civil, criminal, and traffic court obligations. These can include post-arrest, post-conviction, and/or pre-trial issues like unpaid legal debts [15], arrest warrants from failure to appear in court [16], traffic court matters like suspended driver’s licenses, fines, fees, and warrants, as well as civil legal issues, e.g., landlord tenant matters and child support enforcement.

Longitudinal studies by social scientists have identified SUD as a barrier for those trying to reintegrate into society after justice system involvement, largely focused on the criminal justice system (e.g. 17, 18). In particular, individuals with SUD are often referred to treatment through the justice system [2, 19, 20]. Yet incarceration decreases likelihood of recovery from SUD [2, 19]. Less is known about these risks and entanglements on civil court dockets, but similar patterns may hold.

Despite this complex interplay where [1] SUDs increase *risks* of criminal, traffic, and civil justice system entanglements and [2] these entanglements threaten the ability of persons with SUDs to achieve their recovery goals, several gaps in research remain. We first outline these gaps and then discuss the specifics of our randomized controlled trial (RCT) aimed at investigating whether resolving legal issues has a causal impact on improving recovery outcomes.

### Past research on consequences of unresolved legal issues

Past research has focused on the *social and employment* consequences of unresolved legal issues, which can restrict opportunities to participate fully in the local community and economy. Unresolved legal issues can limit a person’s access to healthcare services, employment, housing, and even their own family [21–23]. In contrast, resolving these legal issues is documented to offer improvements in access to housing, employment, and to the family unit, with research demonstrating the value of resolution such as prevailing in unemployment compensation appeals, landlord tenant, family, expungement, social security, and welfare benefit proceedings [24].

Despite the fact that improving access to resources like housing and employment can support treatment completion and long-term recovery [2, 25, 26], little research explores the full recovery pathway of: [1] resolving legal issues, [2] increased access to housing, employment, and other resources, and [3] downstream consequences for an individual’s recovery from a SUD. Qualitative research shows that unresolved legal issues can have a direct impact on clients’ ability to complete clinical treatment regimens, including outcomes like denial of entry to sober transitional housing and interruptions to treatment when clients are revoked to jail.^2^ In addition to arrest warrants, outstanding legal financial obligations (LFOs) incur interest and compounding penalties that can spiral into driver’s and professional license suspensions. LFOs and license suspensions may interfere with one’s ability to work in the formal economy and may spur subsequent criminal activity according to reviews of previous scholarship and correlational research [18, 27–29].

### Need for this randomized controlled trial

Despite prevailing knowledge that treating defendants’ SUD may improve both legal and public health outcomes, there are few policy efforts aimed at improving the *unresolved legal issues* that can serve as barriers to recovery. Some examples of unresolved legal issues include outstanding child support arrears, fines, fees, restitution, and arrest warrants. American criminal and civil justice systems can be intimidating, costly, and difficult to navigate for the average person and more so for those struggling with SUD and those with limited financial resources [18, 30]. We ask: does resolving legal issues have a causal effect on recovery outcomes for individuals with SUD? And if so, what are the potential mechanisms for this causal effect?

To investigate these questions, we partner with a community-based public health organization that delivers care to Medicaid and uninsured clients, and the two community-based non-profit treatment centers who are the primary providers of SUD services for this client population, seven courts, and a tech startup to target online dispute resolution (ODR) to those seeking treatment for SUD to improve treatment outcomes. The ability of our research to capture longitudinal data on treatment outcomes, employment, justice system encounters, housing, as well as family and social support will expand our theoretical understanding of the complex interplay between SUDs, civil and criminal legal system entanglements, and recovery outcomes.

While there are many levers to resolve legal issues—e.g. navigators that accompany clients to in-person court appearances [31]; petition-based expungement [32]; automated expungement [33]—we focus on one type of tool: online dispute resolution (ODR). ODR offers a variety of legal services, remotely, at no charge to defendants to help them address unresolved legal issues.

We focus on ODR based on qualitative research by our team and others on why individuals with SUD may struggle to resolve legal issues. Prior empirical work investigating ODR as a solution to increase access to justice amongst low-income litigants found that many litigants reported they otherwise would not have gone to court to resolve their concerns absent the ODR option [34]. Such avoidance correlates with unresolved legal issues amongst this population. We will overcome this obstacle by reducing or eliminating the need to go into the courthouse with ODR. During the planning and piloting of the online legal platform, focus groups, interviews, and field work reveals that recently sober clients tend to avoid in-person interaction with state agencies, often for five or more years.^3^ This was confirmed with an early participant in our pilot, recruited due to unresolved child support obligations, who had accumulated nearly *two decades* of fines and fees while struggling with SUD. By eliminating the need for face-to-face interactions with the court system, much of the fear, anxiety, and stigma surrounding addiction can be reduced [34, 35]. This particular client successfully negotiated a payment plan, his arrest warrant was vacated, and he is in compliance with his legal obligations for the first time since the 1990s [36].

### Aims and objectives

We focus on the causal impact of an intervention: an offer of hands-on-help with using an ODR tool to resolve legal issues. Our primary aim is focused on the most proximate effect of the intervention: the client’s compliance with goals he or she has set for their own treatment and recovery. We will also explore whether the impact (or lack of detectable impact) on recovery outcomes is accompanied by impacts on more distal outcomes in clients’ ability to secure stable housing, employment, and family reunification.

More specifically, the project intends to answer the following questions:

1. **Primary question**: does offering clients access to and hands-on-guidance with ODR to resolve legal issues increase the client’s likelihood of completing treatment?
2. **Secondary questions**: our ability to investigate these questions depends on data linkages that are still in process. If we are successful obtaining administrative data, we intend to investigate the impact of offers and receipt of hands-on help on the following outcomes, at years one and two following the intervention:
  - Utilization of healthcare/SUD services.
  - Employment.
  - Housing.
  - Family reunification.
  - Mortality.
3. **Heterogeneous effects**: focusing on the primary question, do we see heterogeneous effects across the following subgroups of clients, with attributes measured at baseline:
  - Clients with different personal characteristics/life experiences (e.g., mental illness, trauma history, socioeconomic status, social support).
  - Clients with different manifestations of SUD / patterns of polysubstance use (e.g., opioid use only; opioid use and alcohol; and so on).

## Methods/Design

### Trial design

This is an RCT that involves rolling enrollment of clients over a two-year period. We plan to follow enrolled clients for two years following enrollment and collect administrative data to measure key outcomes in both the treatment and control groups. As discussed in greater detail below, we randomize half of participants to the treatment group, in which they receive access to and hands-on training with an ODR platform. We randomize the other half of participants to the control group, in which they do not receive this intervention. Overall, the project uses exploratory sequential mixed methods design [37–41].

### Participants and clinical setting

The study inclusion criteria are (i) adult (aged 18 years or older), (ii) client receiving direct addiction recovery services at either partnering not-for-profit treatment center, (iii) Medicaid recipient or uninsured.

Participants may be male, female, or non-binary. Participants may be on probation or parole, but do not have to be. People who cannot read or lack computer literacy are eligible and will be offered direct support on these measures from research staff. Participants are offered the research instruments in English and may request a native Spanish speaking research team member and Spanish language research instruments during enrollment.

Two treatment centers are participating in our intervention and have multiple clinical and residential locations throughout Southeast Michigan, including over 180 transitional housing beds scattered throughout the county. Site locations include detoxification, inpatient, outpatient, and transitional housing. All clients are selected from a centralized case management system managed by a local public health agency that serves uninsured and Medicaid clients only. Length of treatment varies from 1 day up to about 2 years. Clients may be referred by friends, family, or law enforcement but also engage services on their own accord. One treatment center partner describes that their referrals often come from “courts, Friend of the Court [child support enforcement], Child Protective Services, Secretary of State, private attorneys, and other service agencies” [19]. Some clients are mandated to treatment in lieu of incarceration and may face incarceration if they fail to comply with the terms of treatment. Clients who participate in standard Drug Court or Sobriety Court typically are ordered to two years of intense supervision by a team of specialists which includes treatment components, but the specified treatment regimen, e.g., detoxification, residential, transitional housing, etc. varies by client.

### Randomization procedure

#### Step 1: flagging potentially eligible clients

Clients who meet the inclusion criteria are *potentially* eligible for randomization. We worked with developers responsible for maintaining the community-based public health center’s centralized case management system called CRCT/Cricket (Confidential Record of Consumer Treatment) that is overseen by a community-based public health organization. These developers created a set of decision rules that randomize clients at intake who both meet the above *person-*level eligibility criteria and meet the following *visit-*level eligibility criteria. Since the *same person* might have *multiple visits* to the participating treatment centers, this visit-level eligibility criteria help ensure that those with multiple visits remain in the group tied to their *first intake* within the study window, as opposed to being re-randomized to potentially different groups each time. More specifically, the visit-level rules are:

1. A new record has been created for the client.
2. The client has not already been enrolled in the study (to prevent clients who return to one of the participating treatment centers multiple times from receiving repeated “doses”).
3. The client meets other sample criteria (age 18 or older).

Once a treatment client has been identified as *eligible for randomization*, they are invited by research staff to then consent to participation in the study. Clients are provided a release of information consent by research staff, affording researchers permission to speak to them as their identity as a recipient of SUD services is protected. Upon signed release of information consent, researchers provide and go over the study consent form with clients. For clients identifying as having literacy challenges, researchers read the consent and all subsequent written material.

Clients *eligible for randomization* then fall into two groups. For prospective participants who *do not* consent to the study and/or sign the release of information, CRCT removes them from the study’s enrollment tables. For prospective participants who *do* consent to the study *and* sign the release of information, they proceed to the randomization step. Participants can discontinue participation at any time by declining consent or revoking consent and notifying the PI they wish to revoke consent. Assignment to the intervention is not blinded.

#### Step 2: randomizing clients who consent

Upon a client’s signed release of information and consent forms, they are eligible for randomization. Logic within the CRCT system randomizes the participants to one of two groups with 0.5 probability: the treatment group or the control group. Due to the limited fields in CRCT available at intake, we did not conduct stratified randomization. Each group then proceeds to their respective intervention.

#### Step 3: treatment group participants receive the intervention

#### Step 4: both treatment and control group participants complete the life-course history survey

We invite participants in both groups, using a standard script, to complete our life-course history survey measuring demographics, polysubstance use, mental health, and several other topics discussed in greater detail later.

### Treatment intervention: hands-on-help with ODR platform software

Here we describe the two components of the treatment intervention. First is the platform software itself. Second is the hands-on-help navigating this platform. Based on our focus groups, each component is potentially important for addressing the needs of those with SUD.

#### Component one: ODR platform software

The tech start-up Court Innovations has [7] software tools offered in fifty-one districts throughout Michigan. The software resolves what are considered “minor” legal disputes, but among the substance using community these minor legal disputes can present major obstacles to participating fully in recovery, as well as with obtaining housing, a job, or reuniting with family. Software tools include Warrants, Amnesty, Friend of the Court (child support enforcement), Driving While License Suspended, Online Plea, and Ability-to Pay. The online platform allows clients to communicate with judges, clerks, and prosecutors remotely (at the court’s discretion), complete forms, upload, and sign documents for the court, and resolve outstanding legal obligations electronically, including vacating warrants without ever stepping foot in the courthouse. Court officials offer the client a resolution or request more information. The process is streamlined in a user-friendly online forum that can be accessed from any mobile device. Documents may be uploaded via smartphone photos.

The software has the following features that might be especially useful for those in treatment for SUDs:

- **Reducing stigma felt during face-to-face court appearances**: reducing the need to come into court to describe one’s substance misuse in public can be extremely valuable and destigmatizing. Personal dialogue has been demonstrated to be easier to share over an online platform than in front of community members in open court [34, 35].
- **Access during all hours rather than just court hours**: The platform is accessible 24 hours a day, seven days per week and courts generally respond within three days or less [34].^4^ The 24-hour a day access can be important for clients whose residential treatment makes workday court appearances challenging.
- **Mobile friendly**: The software is intended as a user-friendly online forum that can be accessed from any mobile device. Documents may be uploaded via smartphone photo. Research staff can facilitate mobile upload on research team devices or help facilitate permission and access for clients to utilize their own mobile device for this purpose. This eliminates the need for clients to track down computer equipment like a scanner, which may be unavailable to them during clinical treatment. The supportive nature of the intervention and mobile capabilities help overcome barriers for many clients who otherwise have no or limited access to mobile devices while in treatment.

While these features of the ODR software are promising and some recent scholarship has found evidence that ODR and virtual court proceedings, which carry similar features to ODR—namely not having to go into the courthouse—can improve access to justice for vulnerable clients [34, 42], other scholarship found that technologies can *widen* rather than *reduce* inequalities if they are not straightforward to use for marginalized populations [43]. Therefore, we view the software alone as a necessary but not sufficient intervention for resolving legal issues among those with SUDs. We now turn to the second component, which is meant to help make the ODR software accessible to participants.

#### Component two: hands-on-help with the ODR platform software

The second component for treatment group participants is hands-on help with the ODR software. Participants in this group will be introduced to ODR and given staff assistance in utilizing the tool for online legal resolution. Participants lacking technological literacy will be offered direct support from research staff who will walk them through the web content and read all electronic content to the participant.

### Control group

Participants randomized to the control group could technically access the ODR software, which is free and available to members of the public. Yet to do so, they would [1] need to seek out the platform on their own and [2] navigate the technological barriers to using the software for unresolved legal issues. Our fieldwork in treatment centers shows that we expect low control group uptake of the software due to inpatient clients’ lack of access to technology, low or limited levels of technology literacy for many clients in our participant demographic, combined with low literacy levels reported among many clients, and lack of knowledge that this resource exists or how to navigate it successfully. While inpatient, clients have very limited access to phones and computers, and internet access is generally limited to scheduled and monitored supportive services with a staff member present, such as completing an application for food assistance. Approximately 1 in 3 clients identifies as having literacy difficulties, either with reading, with technology, or a visual impairment that inhibits their ability to access and utilize a web browser without a support person. Finally, like many public resources, uptake rates can be limited among vulnerable populations, who may not know the resource exists and have barriers to accessing it successfully.

### Sample size justification

We intend to enroll 800 participants, with half of the clients randomly assigned to treatment (hands-on training with free ODR) and the other half randomly assigned to the control group (no ODR). A list of final study site locations may be requested from the primary investigator (PI). To determine whether we are sufficiently powered to detect an effect, we conducted a power analysis focused on the primary outcome: the client’s compliance with the treatment plan. We calculate the sample size needed for the study by applying the significance level of 0.05 and 80% power for a two-sided test. Since we lack both site-specific base rates of compliance and data on the possible effect size of hands-on training with ODR, we analyzed power along different dimensions of [1] base rates of treatment plan compliance in the control group and [2] possible improvements in compliance from the hands-on training with ODR. We set cases when the control group’s base rate of the treatment plan compliance ranges from 0.3 to 0.8 and then estimate the sample size necessary when the treatment group turns out to show 2–15% improvement from the control group. From this calculation, we find that 712 participants are necessary to find the difference between the compliance rate of 30% and 40% between the control and the treatment group. When the base rate of treatment plan compliance is 50% for the control group and 60% for the treatment group, we need 775 participants to find the distinction. If the compliance rate for the control group is 80%, 398 participants can inform us of the 10% difference between the two groups. Based on the result, we conclude that 800 participants allow us to detect a ~ 10% point improvement or larger; if compliance rates are high, we can detect much smaller improvements.

### Study outcomes

The study outcomes in both groups will be measured at baseline (T0) and at one year (T1) and two years (T2) after baseline. The primary outcome will be substance use behaviors, which we use electronic medical records data to measure. Secondary outcomes measured with administrative data include (i) mortality, (ii) employment and earnings, (iii) justice system involvement, e.g., civil and criminal legal system encounters, (iv) housing status and household composition.^5^Table 1 shows the SPIRIT flow diagram for the schedule of enrollment, intervention and assessment.

**Table 1:** SPIRIT diagram for the schedule of enrolment, interventions, and assessments

	Study period				
	Enrolment	Allocation	Post-allocation	Post-allocation year 1	Post-allocation year 2
Timepoint	- t1	0	T0	T1	T2
Eligibility	X				
Informed consent	X				
Allocation		X			
Interventions:					
Intervention group			X		
Control group			X		
Assessments:					
Primary Outcome			X	X	X
Secondary Outcome			X	X	X

The primary outcomes of interest are substance use behaviors. The secondary outcomes of interest are (i) mortality, (ii) employment and earnings, (iii) justice system involvement, e.g., civil and criminal legal system encounters, (iv) housing status and household composition (see Footnote 5)

The study outcomes will be measured at baseline (T0) and at year 1 (T1) and year 2 (T2) for both groups

### Data sources

#### Survey for baseline attributes

In-depth life course history online surveys will be administered to all consenting clients at both centers who meet sample criteria at intake. Surveys will be completed electronically on secure servers using Qualtrics. Both treatment and control samples complete the life course history survey. Participants are compensated with a one-time $30 Visa gift card incentive. The survey instrument improves reliability and validity by utilizing published peer-reviewed scales known to be reliable measures. Moreover, original measures were developed, evaluated, and improved utilizing participant-based design that included people who use drugs, SUD clinicians, and persons in recovery from SUD as question writers, focus group participants, and survey testers. The survey asks about past drug and alcohol use patterns, mental and physical health, employment, housing, family arrangements, outstanding legal issues and includes the ACE (Adverse Childhood Experiences) scale, Kessler K6 scale, and peer-reviewed questions from instruments utilized in the Boston Reentry Study [14]. The ACE instrument, which is a widely accepted measure of childhood trauma predictive of adult adversity, is used to evaluate additional barriers related to recovery and increased propensity towards substance misuse [44]. The K6 Kessler scale is utilized to detect moderate mental distress. Survey data will be used to form retrospective life history reports. With these rich data we can better isolate correlations between background and recovery and understand who struggles most with substance use and legal issues and what their triggers are. We can also examine baseline equivalence between participants randomized to the treatment and control groups. Consent and survey documents are available upon request to the PI.

#### Court Innovations user data to measure engagement with ODR

Court Innovations is an ODR tech startup which originated at University of Michigan Law School and provides the justice system platforms utilized in this research, which operate directly with courts, without lawyers. Court Innovations has agreed to share user data with us from clients consenting to participate in our research. We will obtain Court Innovations data for our participants each year following enrollment for two years. These data will offer search statistics, so we know who searches for a case and when, if they officially make a request to begin a resolution with the court, and the disposition of the resolution with all communications between litigants and the courts. As discussed later, it will also help to determine [1] which participants randomized to the treatment group never utilized the software despite the hands-on training and [2] if any participants randomized to the control group utilized the software (e.g., through knowledge sharing between treatment group clients in clinics and their control group counterparts).

#### Treatment case files to measure treatment plan compliance

Primary outcomes are collected from electronic medical records. When consenting to participation, clients will be asked if they consent to us reviewing their treatment center electronic medical records during our intervention and two-year follow-up period. Treatment centers collect comparable metrics center to center because of local, state, and federal requirements and collaboration with Community Mental Health for Southeast Michigan, a primary funding and support resource for uninsured and Medicaid eligible persons diagnosed with SUD. Client demographics, contact information, substance use history, housing status, e.g., homeless, transitional housing, etc., mental health diagnoses, compliance with treatment, participation in 12-step programs, drug and sobriety court involvement, drug and alcohol histories and preferences, and when applicable, date of death will be examined.

#### Potential additional data sources for secondary outcomes

In addition to the primary outcome, we will seek out administrative data on wages and employment status (Michigan Unemployment Insurance Agency), public benefit use, e.g., amount and duration (Michigan Department of Health and Human Services), and criminal justice interactions, e.g., charges, time served, community corrections, probation and parole interactions (State Court Administrative Office), so long as we continue the research. We will also apply to the National Center for Health Statistics (NCHS) National Death Index to collect mortality data for using the names, social security numbers, and/or birth dates of clients lost to follow up [45]. Clients will be asked to consent to administrative data linking. Records will not be linked without consent.

We will also supplement the administrative data with web scraping to collect additional data on mortality, also conducted with the consent of project participants. In particular, while the National Death Index (NDI) provides authoritative mortality statistics, there is a three-year lag where deaths in the year 2020 will not be searchable in the NDI until 2023, for instance. To provide more real-time measures of what has happened to clients that treatment centers have lost contact with, we will use web scraping focused on two types of data. First, is data that can help confirm a participant is alive but has potentially left Michigan. One easily accessible set of data is jail websites that post information on bookings [46]. We will conduct annual scrapes of the booking pages of jails in five neighboring states: Wisconsin, Illinois, Indiana, Ohio, and Pennsylvania. Second is data that can provide a more rapid estimate of mortality than the NDI: online notices of deaths in local newspapers, house of worship bulletins, legacy.com, and other sources. Other researchers have validated the use of online death notices against administrative records of deaths [47, 48]. Boak et al. [47], focused on Pittsburgh, found ~ 70% of deceased persons in the online notices. While the stigma of addiction related deaths may make online death data less reliable for this population, combined, the two sources can provide probabilistic estimates of what happens to participants whom treatment staff no longer are in contact with and who no longer appear in the Michigan administrative data sources.

### Statistical methodology

We will summarize baseline and demographic characteristics using means and standard deviations (or interquartile ranges and medians) for continuous variables and percentages for categorical variables. We will report the proportion of eligible clients consenting to participate and other descriptive data on outcomes such as treatment compliance rates.

For identifying the causal impacts, our main analysis within the RCT will focus on two estimands. First, we will analyze outcomes among the intent-to-treat (ITT) sample, or those offered access to ODR regardless of whether they use the tool or have an eligible case. Because unobserved features of clients—for instance, their tech-savviness or how pressing their legal issue is—impact how clients use the tool, this provides the strongest causal estimate of the tool’s effect. Second, we will analyze outcomes among the “opt in and eligible sample”(TOT), which represents the effect of the assistance + tool on those who actually used the tools to resolve legal issues.^6^ This treatment-on-treated estimand is relevant for policymakers’ interpretations of the value of ODR for substance users—it corresponds to the counterfactual, “what would happen if we adjust the estimates to assume that we are able to tailor the offers to those who will actually use the tools and who have eligible cases to use them for?”

Our estimation approach will depend on the specific outcome variable in question and will be specified in greater detail in a later pre-analysis plan. Broadly, for the intent-to-treat “ITT,” we will examine using non-parametric differences in means (since covariates, in expectation, will be balanced across the groups due to the randomization) and linear and logistic regression, where the coefficient of interest is one on a binary variable for offer of treatment [49]. For the treatment-on- treated “TOT,” we will use the two-stage least squares approach where we regress our measure of compliance (“uses the ODR”) on the offer of the legal tool, and then use the fitted values from step one to measure the impact on outcomes. We will also use survival models for time-dependent outcomes (e.g., time to relapse; time to recidivism; time to mortality).

## Discussion

People with SUD are disproportionately engaged in the justice system. Justice system involvement can create tangible obstacles to completing treatment for SUD. The present RCT advances knowledge by assessing the efficacy of ODR to improve treatment outcomes for persons in treatment for substance use. Our RCT studies these issues both generally and among individuals who are disproportionately impacted by both the justice system and SUDs. In particular, African American and American Indian Alaska Native persons are disproportionately justice-system involved in the United States [50], known to be at heightened risk for premature mortality due to substance use and related causes [51], and are notably understudied in traditional clinical trials [52]. Little research has both focused on targeted legal interventions for persons seeking treatment from SUD and designed the study to allow investigation of disparities amongst African American and American Indian Alaska Native persons.

In addition to the study’s substantive findings, the RCT also develops a novel longitudinal dataset of persons suffering from substance use disorder. The archived deidentified data will permit and encourage researchers from multiple fields to analyze not only the epidemiological outcomes of substance use disorder, such as mortality, but to engage with rich contextual data on the socio-legal circumstances facing the population—specifically among the realms of employment, law, housing, and family. Advancing our knowledge will improve our understanding of the acute socio-legal needs faced by individuals with substance use disorder and develop recommendations to help target resources towards the realms which best support long-term recovery from substance use.

The dataset will be deidentified and issued open access for public use in addition to academic inquiry. If the RCT finds a tangible benefit of targeting ODR to treatment centers, evidence of this benefit can be disseminated broadly so that individuals with substance use disorder might access ODR to improve treatment outcomes nationwide. Not only can knowledge created from this research potentially improve the well-being of those suffering from substance use disorder, but also their family unit. Spouses, parents, and children who may experience harm from family members’ addiction absent an intervention, might experience reduced family financial burdens, lower levels of state involvement such as foster care, or decreased risk of family violence if treatment completion rates improve. Moreover, a broader understanding of the socio-legal context of the substance dependent population can inform legislators and government to help target limited resources towards high-need policies which support long-term recovery from substance use.

### Limitations

This intervention will be conducted in English or Spanish. The applicability of the study findings to persons not speaking English or Spanish is unknown. The intervention is administered in the Midwest among the uninsured and Medicaid consumers. The applicability of the study findings in other regions is uncertain, though we suspect that benefits may be similar for other uninsured and Medicaid consumers throughout the US.

## Data Availability

Data will be de-identified and made publicly available online. Per IRB, only approved faculty have access to unique identifiers for up to ten years for data analysis purposes. To request data from this study, please contact the PI, Meghan O’Neil.

## Footnotes

1 In 10–30 year follow-up of those who do not experience death.

2 Throughout, we use the terms “client” and “participant” to refer to patients in treatment for SUD.

3 Focus groups 8/24/2019 at Dawn Farm Treatment Center, 8/28/2019 at Court; interview with jail sobriety staff leader 8/23/2019; interviews with Clinical Director of Home of New Vision 7/23/2019 & 8/21/2018; interview with Dawn Farm Recovery Support Specialist and Housing Manager 2/15/2019, interview with Clinical Director of Dawn Farm 5/21/2019.

4 In judicial interviews, court administrators and judges reported that the Court Innovations interface provided them a user-friendly live reminder (like a counter on their desktop) of how long their cases were open and this prompted swift resolution with most trying to respond in two days or less of the litigant’s request being initiated. Judges also noted that resolutions were faster to achieve using Court Innovations relative to standard in-court procedures, so they remained motivated to act expeditiously and keep the momentum going.

5 For the purposes of this protocol, we have omitted those secondary outcomes. Prior to analysis of any linked treatment-outcomes, we plan to post a pre-analysis plan that specifies the exact operationalization of each outcome and data sources for each.

6 Our pre-analysis plan will specify the exact definition of “treatment take up,” which might be defined either by reports by those implementing the hands-on assistance or through data from the ODR software.

## Abbreviations

NSF: National Science Foundation
NIH LRP: National Institutes of Health Loan Repayment Program
RCT: Randomized controlled trial
SUD: Substance use disorder
LFO: Legal financial obligations
GDP: Gross domestic product
ODR: Online dispute resolution
ACE: Adverse Childhood Experiences
PI: Primary investigator
